# Multi-Center Fetal Brain Tissue Annotation (FeTA) Challenge 2022 Results

**DOI:** 10.1109/TMI.2024.3485554

**Published:** 2025-03-17

**Authors:** Kelly Payette, Céline Steger, Roxane Licandro, Priscille de Dumast, Hongwei Bran Li, Matthew Barkovich, Liu Li, Maik Dannecker, Chen Chen, Cheng Ouyang, Niccolò McConnell, Alina Miron, Yongmin Li, Alena Uus, Irina Grigorescu, Paula Ramirez Gilliland, Md Mahfuzur Rahman Siddiquee, Daguang Xu, Andriy Myronenko, Haoyu Wang, Ziyan Huang, Jin Ye, Mireia Alenyà, Valentin Comte, Oscar Camara, Jean-Baptiste Masson, Astrid Nilsson, Charlotte Godard, Moona Mazher, Abdul Qayyum, Yibo Gao, Hangqi Zhou, Shangqi Gao, Jia Fu, Guiming Dong, Guotai Wang, ZunHyan Rieu, HyeonSik Yang, Minwoo Lee, Szymon Płotka, Michal K. Grzeszczyk, Arkadiusz Sitek, Luisa Vargas Daza, Santiago Usma, Pablo Arbelaez, Wenying Lu, Wenhao Zhang, Jing Liang, Romain Valabregue, Anand A. Joshi, Krishna N. Nayak, Richard M. Leahy, Luca Wilhelmi, Aline Dändliker, Hui Ji, Antonio G. Gennari, Anton Jakovčić, Melita Klaić, Ana Adžić, Pavel Marković, Gracia Grabarić, Gregor Kasprian, Gregor Dovjak, Milan Rados, Lana Vasung, Meritxell Bach Cuadra, Andras Jakab

**Affiliations:** Center for MR Research, University Children’s Hospital Zurich, University of Zurich, 8006 Zürich, Switzerland; Department of Perinatal Imaging and Health, King’s College London, SE5 9RS London, U.K.; Center for MR Research, University Children’s Hospital Zurich, University of Zurich, 8006 Zürich, Switzerland; Neuroscience Center Zurich, University of Zurich, 8057 Zürich, Switzerland; Laboratory for Computational Neuroimaging, Athinoula A. Martinos Center for Biomedical Imaging, Massachusetts General Hospital/Harvard Medical School, Boston, MA 02114 USA; Computational Imaging Research Laboratory, Department of Biomedical Imaging and Image-Guided Therapy, Medical University of Vienna, 1090 Vienna, Austria; Medical Image Analysis Laboratory, Department of Diagnostic and Interventional Radiology, Lausanne University Hospital and University of Lausanne, 1011 Lausanne, Switzerland; Center for Biomedical Imaging (CIBM), 1015 Lausanne, Switzerland; Athinoula A. Martinos Center for Biomedical Imaging, Harvard Medical School, Boston, MA 02114 USA; Department of Radiology and Biomedical Imaging, UCSF Benioff Children’s Hospital, University of California, San Francisco, San Francisco, CA 94143 USA; Computing Department, Imperial College London, SW7 2AZ London, U.K.; School of Computation, Information and Technology, and School of Medicine and Health, Technical University of Munich, 80333 Munich, Germany; Computing Department, Imperial College London, SW7 2AZ London, U.K.; Department of Engineering Science, University of Oxford, OX1 3PJ Oxford, U.K.; Department of Computer Science, The University of Sheffield, S1 4DP Sheffield, U.K.; Department of Computing, Imperial College London, SW7 2AZ London, U.K.; Department of Engineering Science, University of Oxford, OX1 3PJ Oxford, U.K.; Computer Science Department, Brunel University of London, UB8 3PH Uxbridge, U.K.; He is now with the Institute of Health Informatics, University College London, WC1E 6BT London, U.K.; Computer Science Department, Brunel University of London, UB8 3PH Uxbridge, U.K.; Department of Computer Science, Brunel University of London, UB8 3PH Uxbridge, U.K.; Biomedical Engineering Department, School of Biomedical Engineering and Imaging Sciences, King’s College London, WC2R 2LS London, U.K.; Biomedical Engineering Department, School of Biomedical Engineering and Imaging Sciences, King’s College London, WC2R 2LS London, U.K.; Biomedical Engineering Department, School of Biomedical Engineering and Imaging Sciences, King’s College London, WC2R 2LS London, U.K.; School of Computing and Augmented Intelligence, NVIDIA, Bethesda, MD 20814 USA; Arizona State University, Tempe, AZ 85281 USA; NVIDIA, Bethesda, MD 20814 USA; NVIDIA, Bethesda, MD 20814 USA; Medical Robotics, Shanghai Jiao Tong University, Shanghai 200240, China; Medical Robotics, Shanghai Jiao Tong University, Shanghai 200240, China; Shanghai AI Laboratory, Shanghai 200240, China; BCN-MedTech, Department of Information and Communications Technologies, Universitat Pompeu Fabra, 08002 Barcelona, Spain; BCN-MedTech, Department of Information and Communications Technologies, Universitat Pompeu Fabra, 08002 Barcelona, Spain; BCN-MedTech, Department of Information and Communications Technologies, Universitat Pompeu Fabra, 08002 Barcelona, Spain; Institut Pasteur, 75015 Paris, France; École Polytechnique, 91120 Palaiseau, France; Institut Pasteur, 75015 Paris, France; Centre for Medical Image Computing, Department of Computer Science, University College London, WC1E 6BT London, U.K.; National Heart and Lung Institute, Imperial College London, SW3 6LY London, U.K.; School of Data Science, Fudan University, Shanghai 200437, China; School of Data Science, Fudan University, Shanghai 200437, China; School of Data Science, Fudan University, Shanghai 200437, China; School of Mechanical and Electrical Engineering, University of Electronic Science and Technology of China, Chengdu 611731, China; School of Mechanical and Electrical Engineering, University of Electronic Science and Technology of China, Chengdu 611731, China; School of Mechanical and Electrical Engineering, University of Electronic Science and Technology of China, Chengdu 611731, China; NEUROPHET Research Institute, Seoul 03080, Republic of Korea; NEUROPHET Research Institute, Seoul 03080, Republic of Korea; NEUROPHET Research Institute, Seoul 03080, Republic of Korea; Sano Centre for Computational Medicine, 30-054 Kraków, Poland; Quantitative Healthcare Analysis (qurAI) Group, Informatics Institute, University of Amsterdam, 1012 WP Amsterdam, The Netherlands; Department of Biomedical Engineering and Physics, Amsterdam University Medical Center, 1007 MB Amsterdam, The Netherlands; Sano Centre for Computational Medicine, 30-054 Kraków, Poland; Center for Advanced Medical Computing and Simulation, Massachusetts General Hospital, Harvard Medical School, Boston, MA 02114 USA; Center for Research and Formation in Artificial Intelligence, Universidad de los Andes, Bogotá 111711, Colombia; Center for Research and Formation in Artificial Intelligence, Universidad de los Andes, Bogotá 111711, Colombia; Center for Research and Formation in Artificial Intelligence, Universidad de los Andes, Bogotá 111711, Colombia; School of Electronic and Information Engineering, South China University of Technology, Guangzhou 510642, China; AHU-IAI AI Joint Laboratory, Anhui University, Hefei 230039, China; School of Electronic and Information Engineering, South China University of Technology, Guangzhou 510642, China; School of Electronic and Information Engineering, South China University of Technology, Guangzhou 510642, China; CENIR, ICM, INSERM U 1127, CNRS UMR F7225, Sorbonne Universiteì, 75006 Paris, France; Signal and Image Processing Institute, University of Southern California, Los Angeles, CA 90089 USA; Signal and Image Processing Institute, University of Southern California, Los Angeles, CA 90089 USA; Signal and Image Processing Institute, University of Southern California, Los Angeles, CA 90089 USA; Center for MR Research, University Children’s Hospital Zurich, University of Zurich, 8006 Zürich, Switzerland; Center for MR Research, University Children’s Hospital Zurich, University of Zurich, 8006 Zürich, Switzerland; Center for MR Research, University Children’s Hospital Zurich, University of Zurich, 8006 Zürich, Switzerland; Neuroscience Center Zurich, University of Zurich, 8057 Zürich, Switzerland; Center for MR Research, University Children’s Hospital Zurich, University of Zurich, 8006 Zürich, Switzerland; Department of Neuropediatrics, University Children’s Hospital Zurich, University of Zurich, 8032 Zürich, Switzerland; Croatian Institute for Brain Research, School of Medicine, University of Zagreb, 10000 Zagreb, Croatia; Croatian Institute for Brain Research, School of Medicine, University of Zagreb, 10000 Zagreb, Croatia; Croatian Institute for Brain Research, School of Medicine, University of Zagreb, 10000 Zagreb, Croatia; Croatian Institute for Brain Research, School of Medicine, University of Zagreb, 10000 Zagreb, Croatia; Croatian Institute for Brain Research, School of Medicine, University of Zagreb, 10000 Zagreb, Croatia; Department of Biomedical Imaging and Image-Guided Therapy, Division of Neuroradiology and Musculoskeletal Radiology, Medical University of Vienna, 1090 Vienna, Austria; Department of Biomedical Imaging and Image-Guided Therapy, Division of General and Paediatric Radiology, Medical University of Vienna, 1090 Vienna, Austria; Croatian Institute for Brain Research, School of Medicine, University of Zagreb, 10000 Zagreb, Croatia; Division of Newborn Medicine, Department of Pediatrics, Boston Children’s Hospital and the Department of Pediatrics, Harvard Medical School, Boston, MA 02115 USA; Medical Image Analysis Laboratory, Department of Diagnostic and Interventional Radiology, Lausanne University Hospital and University of Lausanne, 1011 Lausanne, Switzerland; Center for Biomedical Imaging (CIBM), 1015 Lausanne, Switzerland; Center for MR Research, University Children’s Hospital Zurich, University of Zurich, 8006 Zürich, Switzerland; Neuroscience Center Zurich, University of Zurich, 8057 Zürich, Switzerland

**Keywords:** Deep learning, domain generalization, fetal brain MRI, multi-class image segmentation

## Abstract

Segmentation is a critical step in analyzing the developing human fetal brain. There have been vast improvements in automatic segmentation methods in the past several years, and the Fetal Brain Tissue Annotation (FeTA) Challenge 2021 helped to establish an excellent standard of fetal brain segmentation. However, FeTA 2021 was a single center study, limiting real-world clinical applicability and acceptance. The multi-center FeTA Challenge 2022 focused on advancing the generalizability of fetal brain segmentation algorithms for magnetic resonance imaging (MRI). In FeTA 2022, the training dataset contained images and corresponding manually annotated multi-class labels from two imaging centers, and the testing data contained images from these two centers as well as two additional unseen centers. The multi-center data included different MR scanners, imaging parameters, and fetal brain super-resolution algorithms applied. 16 teams participated and 17 algorithms were evaluated. Here, the challenge results are presented, focusing on the generalizability of the submissions. Both in- and out-of-domain, the white matter and ventricles were segmented with the highest accuracy (Top Dice scores: 0.89, 0.87 respectively), while the most challenging structure remains the grey matter (Top Dice score: 0.75) due to anatomical complexity. The top 5 average Dices scores ranged from 0.81–0.82, the top 5 average 95^th^ percentile Hausdorff distance values ranged from 2.3–2.5mm, and the top 5 volumetric similarity scores ranged from 0.90–0.92. The FeTA Challenge 2022 was able to successfully evaluate and advance generalizability of multi-class fetal brain tissue segmentation algorithms for MRI and it continues to benchmark new algorithms.

## I. INTRODUCTION

In-utero Magnetic Resonance Imaging (MRI) of the fetal brain allows clinicians and researchers to visualize the development of the human brain. The brain development of fetuses can be investigated with MRI starting in the second trimester up until birth, and can be used in fetuses with both typical neurodevelopment and neurological congenital disorders [1]. It can aid in the future development of clinical perinatal planning tools for early interventions, treatments, and clinical counseling, and can be used to explore complex neurodevelopment of different structures within the brain. Large-scale acquisition and analysis of *in-utero* fetal brain MRI requires collaboration from specialized clinical centers as image cohorts of various patient populations tend to be small at each center. A crucial step of analyzing these MR images involves quantifying the volume and morphology of different anatomical structures in the developing brain, necessitating image segmentation. Manual segmentation is time-intensive, susceptible to variability between observers and centers, making it impractical for extensive collaborative efforts. However, many challenges exist where the focus is on developing automatic segmentation tools that will work across data from different imaging centers.

Existing deep learning-based methods work well when they are tested on similar data to which they were trained on (*i.e.* in-domain data), but struggle when facing testing data that is different from the training data (*i.e.* unseen, or out-of-domain (OOD) data), such as images acquired at another site, with a different scanner, or with different scanning parameters [2]. Even after careful image processing, classifiers are able to tell the differences between images acquired with different scanners [3]. Efforts to standardize fetal MRI acquisition parameters across different imaging centers or hospitals have been limited, primarily because fetal imaging relies on specialized sequences that are fine-tuned locally. The appearance of MR images is significantly influenced by various factors, including acquisition parameters, magnetic field strength, MRI coil type, overall imaging setup, and the expertise of the technicians performing the image acquisition. These site differences (or domain shifts) have been shown to be very challenging for deep learning algorithms to handle if there is no similar data in the training dataset [2], [4], [5]. Domain generalizability of automatic segmentation algorithms is an urgent need and is attracting increasing attention in the medical imaging field [6], [7], [8], [9], [10].

In our previous Fetal Tissue Annotation Challenge (FeTA) 2021, we used the first publicly available dataset of fetal brain MRI data to encourage teams to develop automatic fetal brain tissue segmentation methods [11]. However, in this dataset, the training and testing datasets were from the same imaging center. For the FeTA Challenge 2022, we launched a multi-center fetal brain segmentation challenge focused on model generalizability across different imaging centers including two unseen centers.

Here, we describe the multi-center FeTA Challenge 2022 and its organization as well as give an overview of the submitted algorithms and provide a detailed analysis and evaluation of the challenge results. This paper adheres to the transparent reporting guidelines as described in the BIAS method [12].

**Fig. 1.**
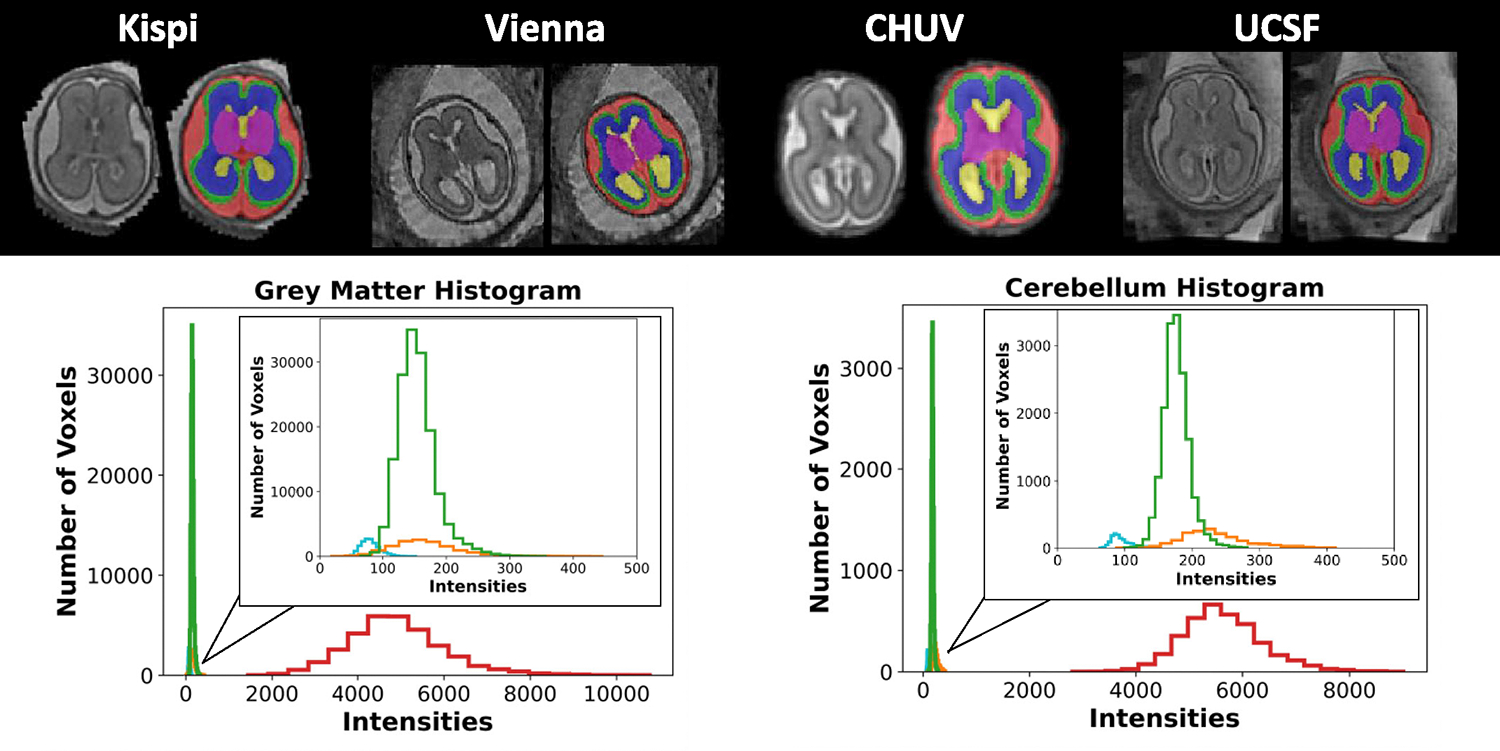
Sample cases from each institution in the testing dataset. Each case is a normally developing fetal brain from gestational week 22, with a super-resolution quality rating of ‘Excellent’. The histograms of the individual labels vary between each institution (green: Kispi, orange: Vienna, blue: CHUV, red: UCSF). The inset is an enlarged view of the first peak to visualize the different histograms of the three institutions.

The aim of the multi-center FeTA Challenge 2022 is to promote the development of domain-robust algorithms for automatically segmenting high-resolution fetal brain MRI reconstructions between 19–35 gestational weeks into seven different classes that works on data from different imaging centers. The challenge includes data from four different imaging centers, further expanding on the FeTA dataset [13]. Two of the centers are included in the training dataset, and all four imaging centers are included in the hidden testing dataset on which the algorithms were evaluated to test on both seen and unseen data. Examples from each site can be seen in Fig. 1. The algorithms are evaluated on the hidden testing dataset. The submitted algorithms are also tested on various subsets of the testing dataset to determine whether they perform better or worse on data from different imaging centers or under different circumstances such as image quality or reconstruction method.

In addition to analyzing the results of the FeTA Challenge 2022, we also propose to investigate the usage of topology as a new evaluation metric for automatic segmentation algorithms. Given that a key downstream analysis of segmentation is the extraction of surface and surface-based metrics (such as thickness and curvature), computational topology of binary masks (*i.e.* connected component, holes) are important to evaluate. We investigate whether topology errors should be added to current evaluation metrics, as challenge evaluation metrics play a significant role in challenge results [14]. Topology holds particular importance for the analysis of the GM segmentation, which remains one of the most challenging structures to segment in the developing brain and requires a topologically correct segmentation for certain analysis, such as determining gyrification.

The algorithms developed as part of the multi-center FeTA Challenge 2022 have the potential to transform both the clinical and research fetal MRI environment, leading to better antenatal and perinatal tools being developed across hospitals and research institutions around the world.

## II. METHODS

### A. Challenge Organization

The FeTA Challenge 2022 (feta.grand-challenge.org) was held in conjunction with the Medical Image Computing and Computer Assisted Intervention (MICCAI) 2022. The challenge was part of a repeated annual event at MICCAI, with a fixed submission deadline. Participants were asked to submit a fully automatic segmentation algorithm that would segment high-resolution fetal brain MRI reconstructions into seven different tissue types: external cerebrospinal fluid (eCSF), grey matter (GM), white matter (WM), ventricles, cerebellum, deep grey matter (deep GM), and brainstem.

In addition to the FeTA training dataset, the participants were able to use additional data for training only if it was publicly available and were required to document the usage in their algorithm description. Participants were able to modify the provided training data as well. This modification includes the generation of additional data by image synthesis or various data augmentation strategies (for example, using numerical simulations by FaBiAN [15]) as long as everything was documented, and the synthetic data could be made available to challenge organizers upon request.

All teams with valid submissions and who presented their results at MICCAI 2022 were included in this paper. Each team was allowed three co-authors. Participating teams were able to publish their algorithms and results independently after the challenge, but should cite this challenge paper and the data publication paper [13].

The full results were announced at the MICCAI 2022 conference and were published on the challenge website. The top three teams received custom-made FeTA chocolate bars as a prize. Participating teams were able to choose whether they wished to make their submission public. The Dockers of all submissions with consent to publicly release can be found here: https://hub.docker.com/u/fetachallenge22. Each team was required to provide a written description of their algorithm, which can be found in the Supplementary Information [16].

Participants were asked to submit a Docker container containing their fully automatic segmentation algorithm to the organizers via email. Members of the organization committee were allowed to participate but were not eligible for awards. The organizers ran the Docker container on the testing datasets using evaluation code available on the challenge website. No multiple submissions were allowed. Resubmissions were only allowed in cases of technical errors with the Docker.

The training dataset was released to participants on June 1, 2022, and the Docker submission deadline was August 3, 2022. The top-performing teams were informed that they were a top-performing team on September 3, 2022, in order for them to prepare a presentation for the day of the challenge. The challenge day was September 18, 2022, where the results were presented at the MICCAI FeTA Challenge 2022 session. For the complete overview of the challenge, see the final challenge proposal [17].

### B. Mission of the Challenge

The mission of the FeTA Challenge 2022 was to encourage and facilitate the development of generalizable automatic multi-class segmentation algorithms that are able to segment the fetal brain into seven different tissue types plus background from MRI. To achieve this goal, clinically acquired, anonymized MRI data were used to represent the target cohort, pregnant women who underwent fetal MRI. The accuracy of the fetal brain segmentations was evaluated in the challenge cohort. Fetal brain MRI scans were acquired clinically and reconstructed using super-resolution (SR) reconstruction methods. The gestational age (GA), and a label of normal neurodevelopment or pathological neurodevelopment was included for each case in the dataset, and the cases spanned a GA range of 18–35 weeks.

### C. Challenge Dataset

**Fig. 2.**
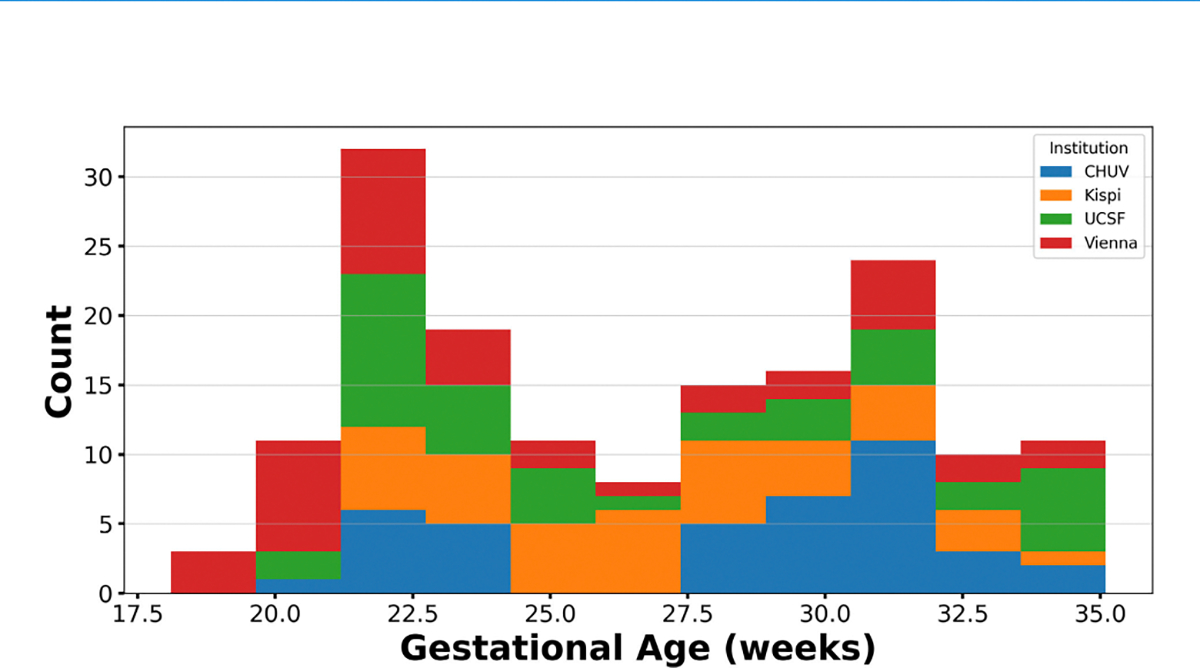
Overview of the gestational ages (in weeks) included within the testing dataset by institution.

The challenge dataset consisted of fetal brain MRI reconstructions acquired from four different imaging centers. Data from two centers (University Children’s Hospital (Kispi), Medical University of Vienna (Vienna)) was included in the training dataset, and an additional two centers were included in the testing dataset (Lausanne University Hospital (CHUV), University of San Francisco (USCF)), for a total of four centers. In this challenge, one case consisted of the following: a SR reconstruction of the fetal brain MRI, a manually segmented label map consisting of eight labels (eCSF, GM, WM, ventricles, cerebellum, deep GM, brainstem, background), a GA, and the classification of normal or pathological neurodevelopment. The testing dataset was hidden from participants. In total, there were 120 cases in the training dataset and 160 cases in the testing dataset (see overview in Table I). Multiples of 40 for the dataset were used as the original FeTA dataset [13] contained 40 cases, and the first FeTA challenge [11] continued this pattern, containing 80 cases. A separate validation dataset was not provided to the participants. The distribution of GAs and the split between normal and pathological neurodevelopment was kept as equal as possible between the two centers included in both the training and testing dataset. For the two unseen imaging centers, a range of GAs, pathologies, and normal neurodevelopmental cases were included to mimic the potential real-world usage of automatic segmentation algorithms. Each case in the dataset was manually segmented using the same method. Several annotators with experience in medical imaging (co-authors (years of experience): AJako (1), MK (1), AA (1), PM (1), GG (1), HJ (5), CS (3), KP (6), AJaka (14)) were trained to segment different labels, and then the individual labels were automatically combined. All segmentations performed by individuals with 1 year of experience were reviewed by the more senior (3 years or more) annotators. Afterwards, three experts in fetal MRI (KP, CS, AJaka) reviewed and corrected each label map, where each case was reviewed by two of the three experts in a two-step process to minimize error. Exact details of the manual segmentation can be found in the supplementary information of [13]. An overview of the GAs included in the challenge’s testing dataset can be found in Fig. 2.

#### 1) University Children’s Hospital Zurich (Kispi) Data:

The training and testing data from FeTA 2021, acquired at the University Children’s Hospital Zurich (Kispi), was used in FeTA 2022, and a detailed description of the image acquisition parameters, post-processing steps, and ethical approval information can be found in [11]. A clinically acquired dataset of 120 brain scans (80 training cases and 40 testing cases) was used as part of the Kispi portion of the FeTA dataset. Several T2-weighted single shot Fast Spin Echo (ssFSE) images were acquired for each subject in all three planes with a reconstructed resolution of 0.5 × 0.5 × 3–5mm^3^. The images were acquired on either a 1.5T or 3T clinical GE whole-body MRI scanners (Signa Discovery MR450 and MR750) without the use of maternal or fetal sedation using an 8-channel cardiac or body coil with the following sequence parameters: TR: 2000–3500ms, TE: 120ms (minimum), flip angle: 90°, sampling percentage 55%. Field of view (200–240mm^2^*)* and image matrix (1.5T: 256 × 224; 3T: 320 × 224) were adjusted depending on the GA and size of the fetus. The data was acquired at the University Children’s Hospital Zurich in Zurich, Switzerland by trained radiographers using clinically defined protocols.

Fetal brain SR reconstructions were performed on the acquired datasets, with a training/testing split of 40/20 using both the mial-srtk method [18], [19] and the simple-irtk method [20]. After reconstruction, each fetal brain volume had an isotropic resolution of approximately 0.5mm^3^, with some deviation in exact dimensions between the SR methods. Each reconstructed image was then histogram-matched using Slicer [21], and zero-padded to be 256 × 256 × 256 voxels. The testing cases were considered in-domain, as this site provides both training and testing cases.

#### 2) University of Vienna (Vienna) Data:

The data from the Medical University of Vienna (Vienna) was acquired using 1.5 T (Philips Ingenia/Intera, Best, the Netherlands) and 3 T magnets (Philips Achieva, Best, the Netherlands), without the use of maternal or fetal sedation. All acquisitions were performed using a five-channel cardiac coil. For each case, at least 3 T2-weighted ssFSE sequences (TE=80–140ms, TR=6000–22000ms) in 3 orthogonal (axial, coronal, sagittal) planes with reference to the fetal brain stem axis and/or the axis of the corpus callosum were acquired. Overall, slice thickness was between 3mm and 5mm (gap 0.3–1mm), pixel spacing 0.65–1.17mm, acquisition time between 13.46 and 41.19 seconds.

The preprocessing pipeline [22] consisted of a data denoising step [23], followed by an in-plane super resolution [24] and automatic brain masking step [25] and concluded with a single 0.5 mm^3^ isotropic slice-wise motion correction and volumetric SR reconstruction [25]. Subsequently, the resulting volumes were rigidly aligned to a common reference space [26].

Fetal MRI cases were provided by the Medical University of Vienna. The data was acquired as part of a retrospective single-center study and was anonymized and approved by the ethics review board and data clearing department at the Medical University of Vienna, responsible for validating data privacy and sharing regulation compliance. There were 40 training cases and 40 testing cases included in the FeTA Challenge 2022 from this site. As with the Kispi data, these testing cases were considered in-domain, as this site provided both training and testing data.

#### 3) Lausanne University Hospital (CHUV) Data:

The data from the Lausanne University Hospital (CHUV) was acquired at 1.5T (MAGNETOM Aera, Siemens Healthcare, Erlangen, Germany), without the use of maternal or fetal sedation. Acquisitions were performed with an 18-channel body coil and a 32-channel spine coil. Images were acquired using T2-weighted (T2W) Half-Fourier Acquisition Single-shot Turbo spin Echo (HASTE) sequences in the three orthogonal orientations (axial, sagittal, coronal); usually at least two acquisitions were performed in each orientation., TR/TE, 1200ms/90ms; flip angle, 90°, echo train length, 224; echo spacing, 4.08ms; field-of-view, 360 × 360mm^2^; voxel size, 1.13 × 1.13 × 3.00mm^3^; inter-slice gap, 10%, acquisition time between 26 to 36 seconds.

For each subject, the scans were manually reviewed and the good quality scans were chosen for SR reconstruction, creating a 3D SR volume of brain morphology [18]. Each case was zero-padded to 256 × 256 × 256 and reoriented to a standard viewing plane. Mothers of all other fetuses included in the current work were scanned as part of their routine clinical care. Data was retrospectively collected from acquisitions done between January 2013 to April 2021. All images were anonymized. This dataset was part of a larger research protocol approved by the ethics committee of the Canton de Vaud (decision number CER-VD 2021–00124) for re-use of their data for research purposes and approval for the release of an anonymous dataset for non-medical reproducible research and open science purposes. As no training cases were included from this site, the 40 testing cases were considered out-of-domain.

#### 4) University of California San Francisco (UCSF) Data:

The data from the University of California (UCSF) was acquired using 3T GE Discovery MR750 or MR750W (wide bore) without the use of maternal or fetal sedation. Acquisitions were performed using a 32 channel GE cardiac coil. At least 3 T2-weighted ssFSE sequences were acquired with one scan per orientation (sagittal, axial, coronal) with the following parameters: 240 mm^2^ FOV with 512 × 512 matrix gives in plane resolution of ~0.5 × 0.5 mm^2^ with 3 mm slice thickness. TR is 2000–3500 ms, TE > 100 ms, 90° flip angle.

For each subject, the scans were manually reviewed and the good quality scans were chosen for SR reconstruction, creating a 3D SR volume of brain morphology [25]. Each case was zero-padded to 256 × 256 × 256 and reoriented to a standard viewing plane.

Fetal MRI was acquired during routine clinical care with institutional review board approval for anonymized retrospective analysis by the FeTA team (IRB 21–35930). As no training cases were included from this site, the 40 testing cases were considered out-of-domain.

### D. Evaluation Metrics

Three complementary types of evaluation metrics were used to compute the rankings. The overlap was quantified with the dice similarity coefficient (DSC) [27]. The similarity between the two volumes was quantified with the volume similarly measure (VS) [27]. The contours were evaluated with a boundary-distance-based metric: the 95th percentile of the Hausdorff distance (HD95) (https://github.com/deepmind/surface-distance). As the task is a segmentation task, the DSC was chosen, as it was the most popular segmentation metric. However, we were not only interested in the overlap, but also the shape and volume, as they are often used as clinical biomarkers. Therefore, we included the HD95 (shape) and VS (volume) metrics. The final rankings took all three metrics into account.

### E. Ranking

The ranking method was the same as in FeTA 2021 [28]. Each of the participating teams was ranked based on each evaluation metric, and then the final rankings combined the rankings from all of the metrics (DSC, HD95, VS) for the complete dataset (both in- and out-of-domain imaging site). The DSC, HD95, and VS were calculated for each label within each of the corresponding predicted label maps of the fetal brain volumes in the complete testing dataset. The mean and standard deviation of each label for all test cases was calculated, and the participating algorithms were ranked from low to high (HD95), where the lowest score received the highest scoring rank (best), and from high to low (DSC, VS), where the highest value received highest scoring rank (best) based on the calculated mean across all labels and test cases. If there were missing results, the worst possible value was used. For example, if a label did not exist in the new segmentation label map but was present in the ground truth (GT) label map, it received a DSC and VS score of 0, and the HD95 score was double the max value of the other algorithms submitted. This ranking procedure was developed to take three different metric types equally into account.

Finally, the results of the challenge were run through the ChallengeR toolkit, specifically designed to calculate and display imaging challenge results [29].

Additional rankings were created based on the in-domain and OOD imaging centers, cases with and without neurological pathologies, and image reconstruction quality (Excellent, Good, Poor). These additional rankings were not part of the determination of the winner of the challenge but were presented at the FeTA Challenge 2022 event.

### F. Topology Analysis

In addition to the rankings mentioned in the previous section, we assessed the topological correctness as an evaluation metric of the predicted label maps. Topology defines the properties of an object that are preserved through deformation [30]. Given binary maps (tissue labels), computational topology relies on connectivity of a voxel to its neighbours to quantify the number of connected components, holes, or cavities. Topology is relevant for exploring brain tissue segmentations as topological correctness is needed to quantify biomarkers important for brain development such as cortical thickness and gyration. However, fetal cortical segmentations (GM) are often discontinuous [31], [32], [33] but surprisingly topology correctness of predicted segmentations is rarely reported [34], [35]. Here, we propose a global topology-integrative ranking (TIR) of the methods, which includes the Betti Number Error (BNE) ranking in addition to the current three evaluation metrics (DSC, HD95, VS).

To quantitatively compare the topology of each segmented structure, we assessed the error of the topological invariant Betti numbers, also known as the BNE. The *k*-dimensional Betti numbers (*BN*_*k*_*)* count the topological structures in each dimension *k.*More *s*pecifically, *BN*_0_, *BN*_1_, *BN*_2_ represented the number of connected components, the number of holes and the number of cavities in the 3D binary object respectively. We define the *k*-dimensional Betti number error (*BN E*_*k*_*)* as the absolute difference of the GT expected value and the prediction measure. *BN E*_*k*_ are difference metrics that must be minimized. The GT expected values are as follows: the *BN*_1_ = 0 and *BN*_2_ = 0 for all brain tissue labels. For eCSF, WM, ventricles, cerebellum, deep GM, and brainstem, *BN*_0_ = 1, and for GM, *BN*_0_ = 2.

When performing the evaluation of the predicted label maps, when there was an absence of segmentation for a tissue, it was attributed twice the value of the worst performing segmentation of the same label over all submissions, in line with how missing data was handled with the HD95 evaluation metric. Once topology was quantified, we also computed the ranking of methods for each *BN E* and TIR ranking with Challenge R toolkit [29].

## III. RESULTS

### A. Challenge Submissions

There were 17 submissions from 16 different teams to the FeTA Challenge 2022. One team submitted two algorithms, but they were determined to be substantially different methodologies and as such was allowed. Each team submitted a written description of their algorithm, which can be found in the Supplementary Information [16]. Two teams used only one institution’s dataset rather than the complete training dataset (deepsynth, ajoshiusc). All other teams used the complete training dataset. Seven teams used additional publicly available datasets for pre-training or training (FIT_1, FMRSK, symsense, FIT_2, DBC Pasteur, fudan_zmic, deepsynth).

**Fig. 3.**
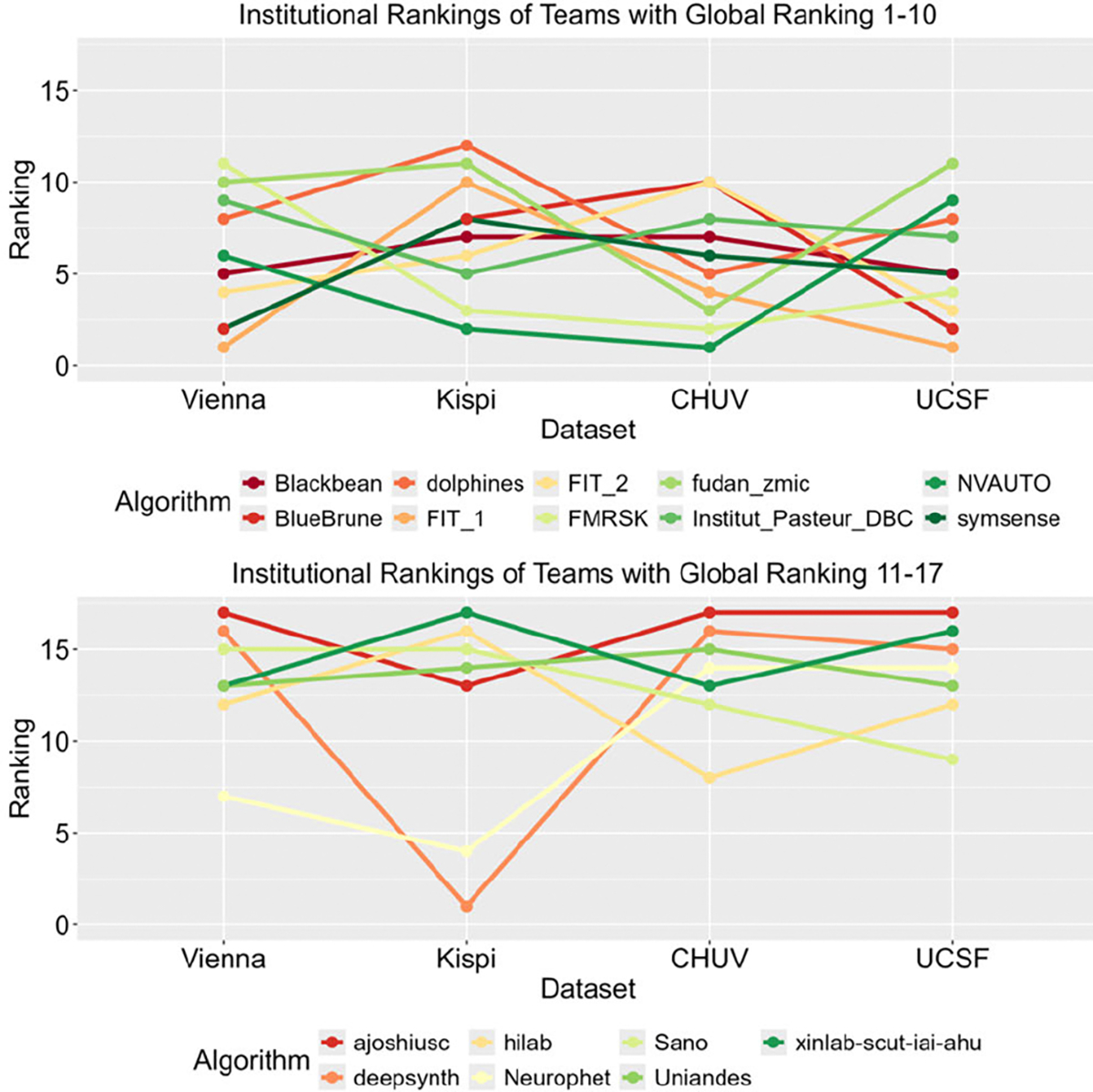
Each submission was evaluated separately on the institutional subsets of the testing data in order to determine if certain algorithms performed better or worse on data from specific institutions. The rankings of the participating teams for each institutional subset are shown, with each connected line corresponding to a single FeTA submission. In-domain institutions: Vienna, Kispi; Out-of-domain institutions: CHUV, UCSF.

All submitted models relied on deep learning. Only three teams used 2D networks (fudan_zmic, DBC Pasteur, ajoshiusc), the remainder of the teams used 3D networks. All teams used PyTorch, or PyTorch-based solutions (such as nnU-Net [36] or MONAI [37]) for their network. Many teams used a two-step strategy for segmentation (often classified as ‘coarse-to-fine’). This often involved first segmenting the brain from the outlying maternal tissue, and then segmenting the fetal brain into different tissues. Each algorithm is summarized in further detail in Table II. Institutional ranking differences in the submissions can be found in Fig. 3.

### B. In-Domain Results

**Fig. 4.**
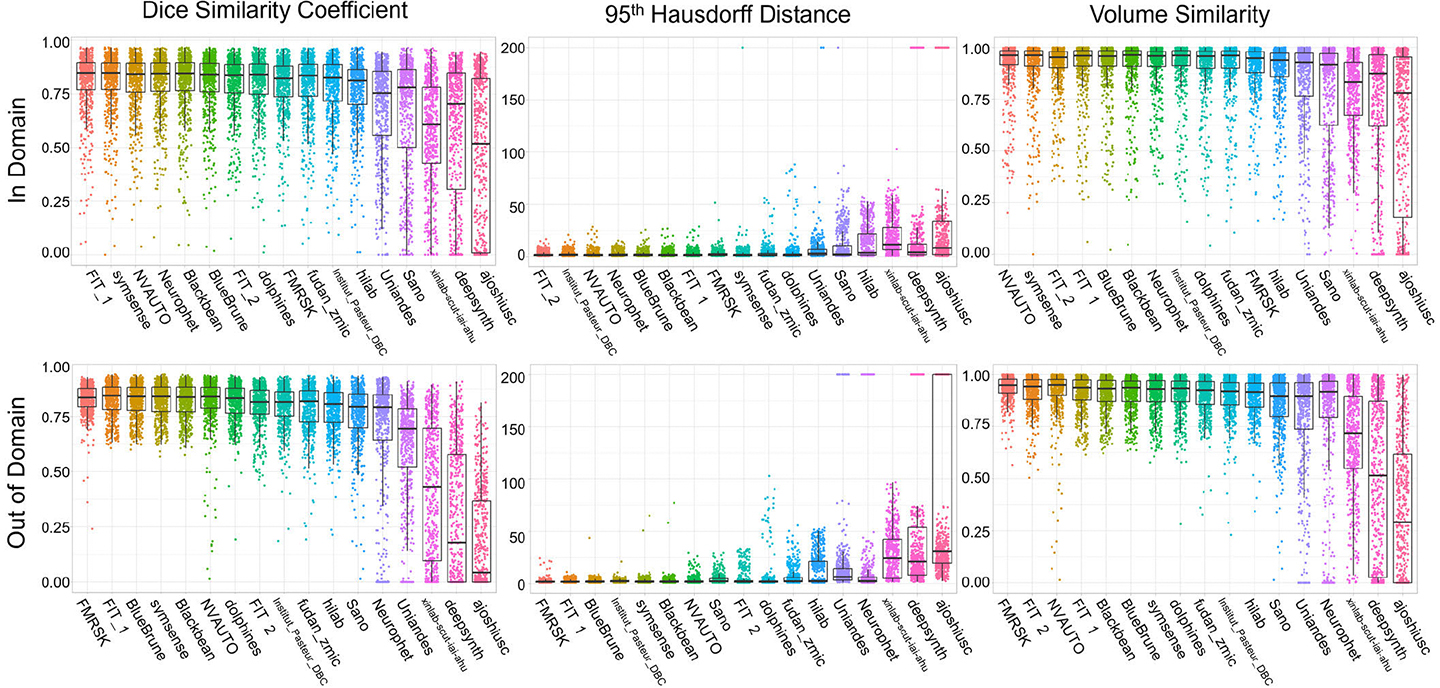
In-Domain and Out-of-Domain evaluation metrics by algorithm. In both in- and out-of-domain, as well as for all three evaluation metrics (Dice Similarity Coefficient, 95th Hausdorff Distance, Volume Similarity), the results plateau for the first 10 teams, after which a drop off is observed. The ranking of the teams has changed between the In-Domain and Out-of-Domain metrics.

In-domain evaluation was defined based on the performance on the subset of data including the Kispi and Vienna data, as data from these two imaging centers were represented in the training dataset available to the participants. A summary of the in-domain evaluation metrics for all teams can be seen in the top row of Fig. 4. We report two aspects of the in-domain evaluation results. Firstly, we present in-domain team rankings and an in-depth evaluation of the FeTA Challenge 2022 results. Secondly, we cross-reference these rankings with the outcomes achieved in the FeTA Challenge 2021 [28]. Notably, the Kispi data included in the FeTA2022 is identical to the FeTA 2021 training dataset (80 cases).

In the overall ranking of the in-domain dataset, the top three submissions were *NVAUTO*, *FIT_2* and *FIT_1*. Specifically, *FIT_1* (0.8052), *symsense*(0.8047), and *NVAUTO*(0.8042) were the top three teams according to the DSC. The top three submissions according to the HD95 were *FIT_2*(2.31mm), *Institut_Pasteur_DBC*(2.40mm), and *NVAUTO* (2.46mm).

The top three submissions according to the VS were *NVAUTO* (0.914), *symsense* (0.910) and *FIT_2* (0.910). It is worth noting that no statistically significant differences were found in the rankings for the achieved DSC scores in the first four teams, (*FIT_1*, *symsense*, *NVAUTO*, *Neurophet*). In the HD95, the first ranked submission, *FIT_2*, was significantly better performing than the second ranked (*Institut_Pasteur_DBC*), while the top two teams in VS (*NVAUTO* and *symsense)* were not significantly different. Further details about the individual rankings are shown in Fig. 4. Similar to the FeTA 2021 Challenge, a performance plateau was observed in the DSC scores, with approximately the first 12 teams achieving very similar DSC scores (DSC range for the top 12 teams: 0.765 – 0.805), with a large drop off in scores in the last five submissions (DSC range for the last 5 teams: 0.455 – 0.684). A similar trend was observed for the mean HD95 (highest ranked 9 submissions: 2.31 to 2.83 mm, lowest ranked 8 submissions: 3.5 to 41 mm) and for the mean VS scores (highest ranked 11 submissions: 0.902 – 0.914, lowest ranked 6 submissions: 0.611 – 0.880).

Not all anatomical structures were segmented equally well, which is reflected by the heterogeneity of mean DSC, HD95 and VS scores obtained in the in-domain evaluation. The WM and ventricles were the structures most successfully segmented. The mean DSC for the top three submissions for the WM were 0.885 (*FIT_1*), 0.883 (*symsense*) and 0.882 (*Blackbean*), and the ventricles were 0.889 (*NVAUTO*), 0.889 (*symsense*) and 0.888 (*FIT_1*). On the other hand, the GM was segmented rather poorly, as the mean DSC for the top three submissions were 0.726 (*FIT_1*), 0.725 (*NVAUTO*) and 0.724 (*Neurophet*). The eCSF spaces, which neighbor the GM, were similarly poorly segmented.

Compared to the FeTA Challenge 2021 results, segmentation accuracy improved marginally. The highest DSC in the FeTA Challenge 2022 in-domain evaluations was 0.805, while it was 0.786 in 2021. The lowest HD95 in the FeTA2022 in-domain evaluation was 2.31 mm, while it was 14 voxels in 2021. These two metrics are not directly comparable due to the change in evaluation tool and unit between the years, as the tool used in FeTA 2021 was not ideal when outliers were present. The highest average VS in the FeTA Challenge 2022 was 0.914, while it was 0.885 in 2021. In-domain, the per-label comparisons yielded similar results: the GM and the eCSF being the most difficult to segment, while the WM and the ventricles were the best performing. There were two teams who submitted to both FeTA 2021 and FeTA 2022 who ranked very well in the in-domain evaluation: *NVAUTO* and *Neurophet*. *NVAUTO* maintained a top in-domain ranking in both years in all three evaluation criteria (2021: DSC 1st place, HD95 1st place, VS 2nd place, 2022: DSC 3rd place, HD95 3rd place, VS 1st place), as did *Neurophet* (2021: DSC 3rd place, VS 5th place, 2022: DSC 4th place, HD95 4th place).

### C. Inter-Site Generalizability Assessment: Out-of-Domain Results

Here, we evaluated the performance of the submissions on unseen datasets (i.e. on data that was not present in the training dataset). Therefore, the OOD performance rankings are presented using the CHUV and the UCSF testing data subset and compared with the in-domain results. A summary of the OOD evaluation metrics for all teams can be seen in the bottom row of Fig. 4.

Some submissions demonstrated equivalent performance for both the in-domain and OOD subsets such as *FIT_1* (ranked 3^rd^ in-domain and 2^nd^ OOD), *Symsense* (ranking 4^th^ for both in-domain and OOD), or Dolphins (ranking 9^th^ for both in-domain and OOD). Interestingly, some methods ranked better in the OOD subset, such as *BlueBrune,* which rose from 6^th^ place in-domain to 3^rd^ place OOD, or *Blackbean* who rose from 7^th^ rank in-domain to 4^th^ OOD. However, some models dropped considerably in performance such as *FIT_2* (from 2^nd^ to 7^th^*)*, *NVAUTO* (from 3^rd^ to 6^th^, performing poorly in many OOD cases, see Fig. 4 bottom row) or Neurophet (from 4^th^ to 13^th^*)*. This indicated that the domain shift present in data from different imaging centers can drastically degrade model performance when being deployed in heterogenous clinical datasets.

Overall, the median performance metrics in the OOD setting remained equivalent to the in-domain, and many of the models attained a plateau of performance around 0.80, 2.5, 0.90 in DSC, HD95 and VS respectively. However, the median of the worst performing methods dropped by a large amount (dropping to approximately 0 for DSC, or 0.25 for VS) while in-domain median performance never reached such low scores (always above 0.50 and 0.75 for DSC and VS respectively for all methods).

Not all brain tissue labels were equivalent when comparing in-domain and OOD results. Class-wise performance (see Supplementary Information, Section 12 [16]) indicated that major drops of performance occur in ventricles (in DSC, HD95, VS), and GM and WM volume (in VS). The achieved performance by top ranking algorithms in the other tissues (eCSF, deep GM, cerebellum, brainstem) were even slightly higher OOD than in-domain (e.g. DSC range of 0.83 to 0.36 OOD while 0.76 to 0.04 in-domain).

### D. Global Ranking

The global ranking was the ranking as defined by using the complete testing dataset from all four imaging centers. The global ranking was the official ranking which determined the winners of the FeTA Challenge 2022.

**Fig. 5.**
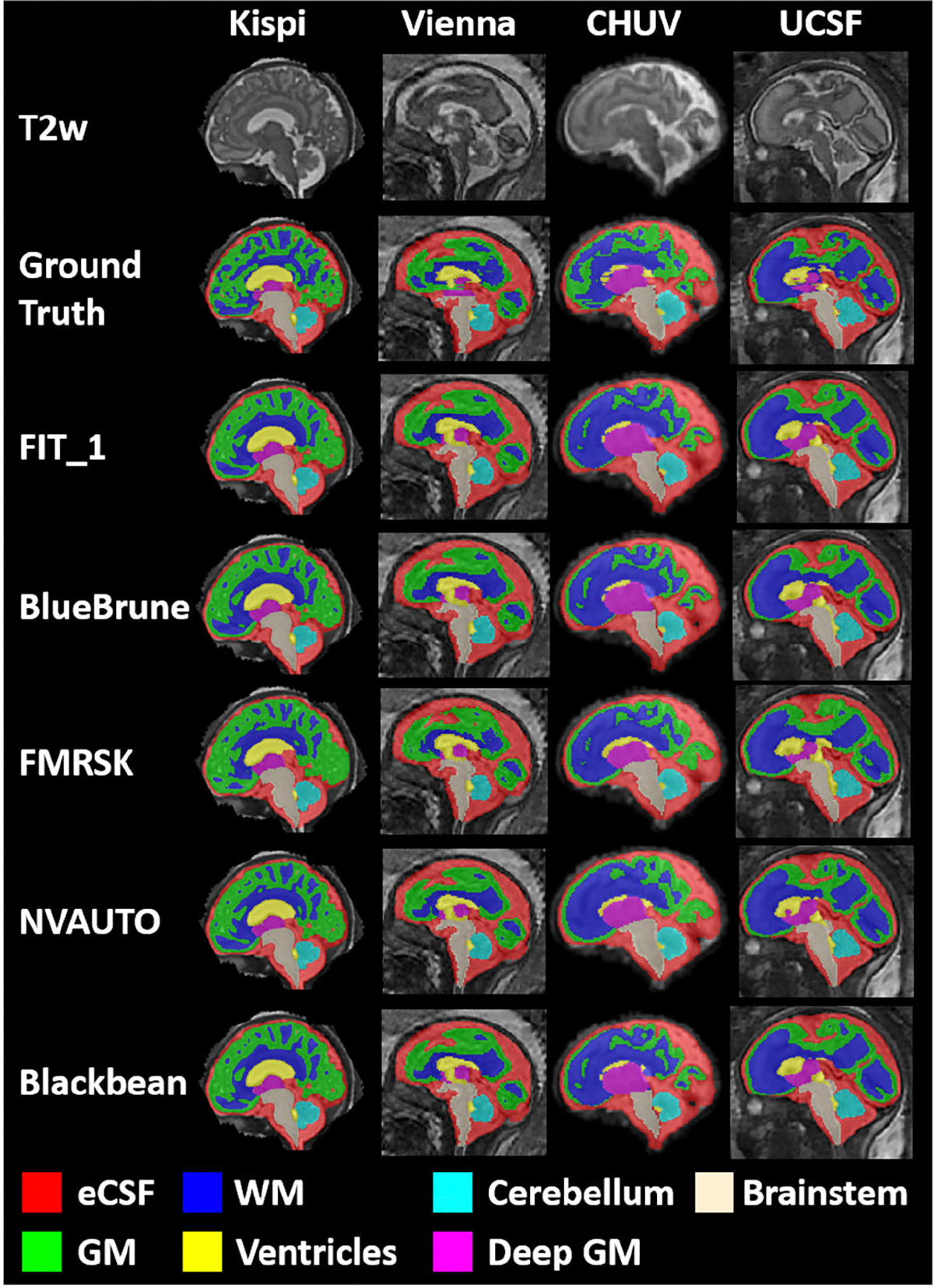
Examples of the automatic labels created by the top 5 teams for each of the four institutions (T2w: T2-weighted fetal brain reconstruction; eCSF: external Cerebrospinal Fluid; GM: Grey Matter; WM: White Matter).

**Fig. 6.**
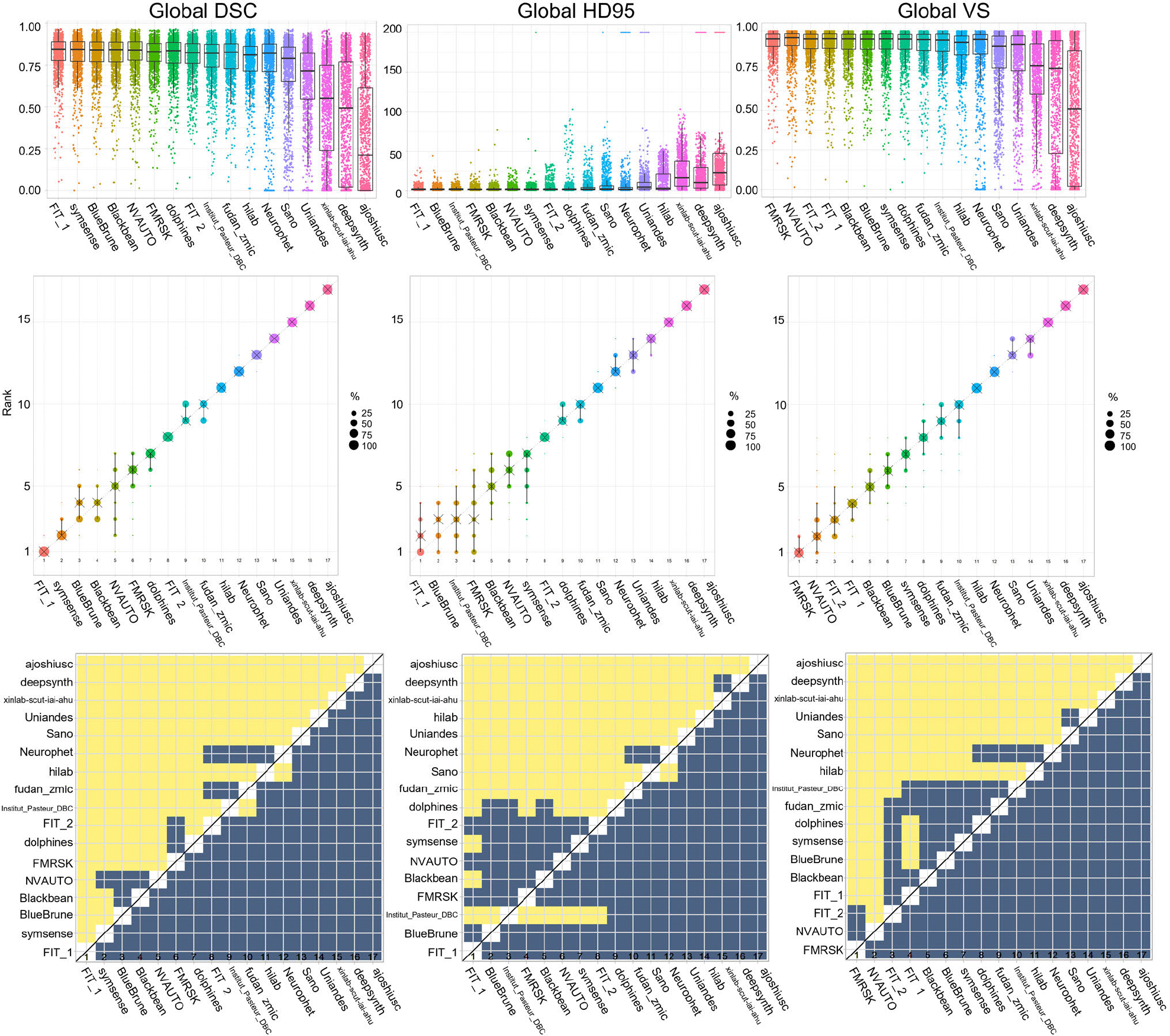
Rankings of participating teams for each metric from top to bottom (left to right). Left column: Global DSC; Middle Column: HD95; Right Column: VS. The first row are box plots of the evaluation data; the middle row visualizes the ranking stability based on bootstrap sampling, and the bottom row displays the significance maps for the ranking stability, where blue cells indicate no significant differences. All plots were generated with the ChallengeR Toolkit. DSC: Dice Similarity Coefficient: HD95: 95^th^ Hausdorff Distance: VS: Volume Similarity.

**Fig. 7.**
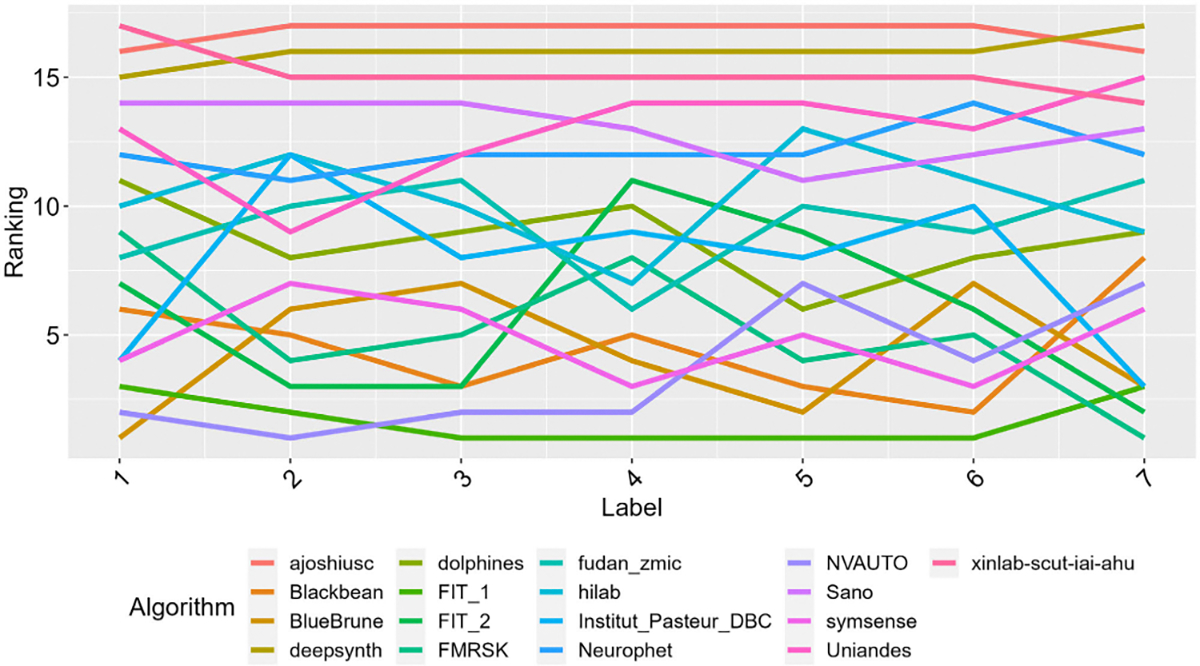
The submissions were evaluated and ranked based on the segmentation results from each of the seven brain tissue labels (1: external Cerebrospinal Fluid, 2: Grey Matter, 3: White Matter, 4: Ventricles, 5: Cerebellum, 6: deep Grey Matter, 7: Brainstem), with each connected line corresponding to a single team’s FeTA submission.

Examples of results from the top 5 teams can be found in Fig. 5. The team rankings of each evaluation metric can be seen in Fig. 6, and the rankings based on the different labels can be found in Fig. 7. The final rankings can be found in Table III.

The top three teams were *FIT_1*, *Bluebrune*, and *FMRSK* (with *Bluebrune* and *FMRSK* tied for second). *FIT_1* maintained a top 5 ranking across each of the labels, while the rankings were variable for all other teams across the different brain tissues. A plateau in the performance of the top 10–12 teams was observed, in line with the in-domain and out-of-domain results. The top three global DSC scores were from teams *FIT_1* (0.816), *symsense* (0.813), and *Bluebrune*(0.812). The top three global HD95 scores were from *FIT_1* (2.35mm), *Bluebrune* (2.38mm), and *Institute_Pasteur_DBC* (2.39mm). The top three global VS scores were from team *FMRSK* (0.920), *NVAUTO* (0.915), and *FIT_2* (0.913).

In order to investigate factors which may have influenced the ratings, we looked at rankings based on quality ratings of the testing dataset (Excellent=3, Good=2, Poor=1, median rating by 3 experienced raters: MBC, AGG, AJaka), normal and pathological brains, as well as rankings based on the SR reconstruction algorithm used (NiftyMIC, mial-srtk, irtk-simple).

For the excellent quality fetal brain reconstructions, the top three teams were *FIT_1*, *FMRSK*, and 4 teams tied for 3^rd^ (*symsense, NVAUTO, Blackbean, BlueBrune*). The ‘Good Quality’ top three teams were *FIT_1*, *FMRSK*, and *NVAUTO*, and ‘Low Quality’ were *BlueBrune*, *NVAUTO*, and *FIT_1*. The top three teams for fetal brains with the normal classification were *FMRSK*, *FIT_1*, and *NVAUTO* and for pathology were *FIT_1*, *BlueBrune*, and *FMRSK*. The top three teams for fetal brains reconstructed with the irtk-simple algorithm [20] were *deepsynth*, *FMRSK*, and *ajoshiusc*; with mial-srtk algorithm [18] were *FMRSK*, *NVAUTO*, and *fudanzmic*; and with the NiftyMIC algorithm [25] were *FIT_1*, *BlueBrune,* and *Blackbean*. When separating the rankings based on individual labels, *BlueBrune* was the top-ranking team for the eCSF, *NVAUTO* ranked first for the GM, *FMRSK* ranked first for the brainstem, and *FIT_1* was the top team for the remaining labels (WM, ventricles, cerebellum, deep GM). A complete overview of the rankings per label can be found in Fig. 7 as well as in the Supplementary Information [16].

### E. Topological Analysis Results

Table IV (A) presents the BNE rankings of the submissions for each dimension *k* ∈ {0,1,2} and the global TIR. The topology rankings were similar across the three *k*-dimensional BNEs, with a maximum rank difference of less than three with one exception: *FMRSK* presented a relatively big change in its BNE rankings of dimension 1 (rank=4) and 2 (rank=13). Potentially, such inter-dimension variation may come from tissue-specific errors. Interestingly, *hilab,* which does not perform well in *BN E*_0_ (rank = 10) and *BN E*_1_ (rank = 11), is the best performing submission in *BN E*_2_ (rank=8). Nonetheless, the good *BN E*_2_ performance was not sufficient to pass on to the global BNE ranking.

Changes in the TIR (see Table IV(B)) were small compared to the global challenge ranking without topology, with a maximum of one rank difference, with one exception. Team *Blackbean* moved from rank 5 in the standard global FeTA ranking to rank 3 in the TIR. The winner and second-place teams remained the same.

**Fig. 8.**
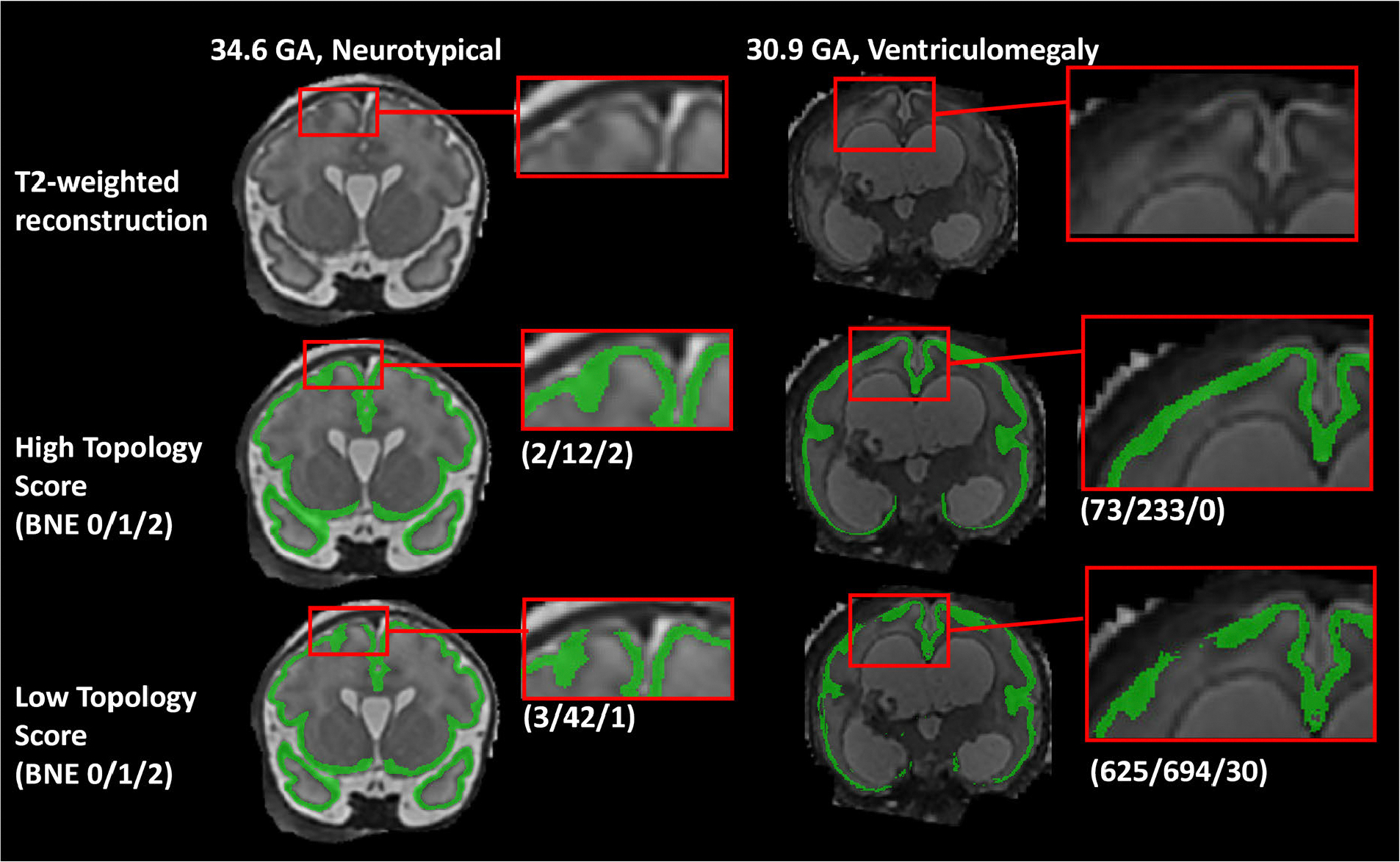
Examples of high and low topology scores of the grey matter segmentations in the challenge results. Left Column: Coronal view of a 34.6 GA fetus with no known neuropathology; Right Column: a 30.9 GA fetus with severe ventriculomegaly and other abnormalities. In the segmentations with high topology scores a continuous cortical ribbon can be observed. In cases with low scores, a gap in the grey matter can be observed in both examples, as well as holes in the segmentation in the ventriculomegaly example, leading to poor scores (where higher numerical BNE scores correspond with poorer results). A perfect BNE score for the cortical grey matter is (2/0/0). The corresponding Betti numbers are displayed in parentheses next to each example. GA: Gestational Age; BNE: Betti Number Error.

Table V presents the global topology BNE ranking of the submissions per tissue class. The TIR of the individual tissues varied within each team’s algorithm. For instance, *hilab* ranked first for the eCSF, but 13^th^ in the WM. Examples of good and bad topology results in the GM can be found in Fig. 8

Apart from the top 2 teams according to topology (*FIT_1* and *BlueBrune*), only *Blackbean* and *Dolphins* were ranked in the upper half for all tissue class. The average tissue TIR of *Blackbean* was 3.3, while *FMRSK* (who tied for second place in the global FeTA ranking) ranked on average 9.1 based on the individual tissue BNE rankings.

## IV. DISCUSSION

The practical value of MRI segmentation methods in clinical settings depends on their ability to effectively generalize to previously unseen data. MRI acquisition settings and various post-processing methods, including image reconstruction, may increase differences between images of the developing fetal brain across imaging sites. Additionally, the overall image quality tends to be lower in comparison to MRI scans of the adult human brain, leading to less distinct delineation of anatomical structures.

### A. Generalizability of Submitted Algorithms

Our results have shown that generalizability across multiple sites remains a challenge for fetal brain MRI segmentation, but resources such as multi-site datasets have the potential to improve the performance of such methods. For example, the top scoring team of the Kispi dataset (ajoshiusc) did not train on the second site’s available training dataset, and subsequently performed poorly on the data from the three additional sites, leading us to assume that this network was overfitted. For some methods (but not all), there seemed to be a preference for a given SR method in the rankings. The winning team (*FIT_1*) ranked first in the Vienna and UCSF datasets, which were both reconstructed with the NiftyMIC SR algorithm. Teams *NVAUTO* and *FMRSK* performed similarly well on the CHUV and Kispi dataset, which included reconstructions performed using the mial-srtk SR method.

Our findings further indicate that image augmentation is a critical factor in achieving good domain generalization. Traditional techniques (i.e. affine transformations, contrast adjustments) have demonstrated their effectiveness within established segmentation frameworks, including nnU-Net. However, the optimum choice of augmentation techniques remains unclear. As highlighted in Table II, it is noteworthy that the leading teams, especially *FMRSK* and *FIT_1*, utilized random bias field and motion artifact augmentations (such as MR spikes). Such data augmentation strategies are specific to MRI images and can mimic potential real-world image differences between scanners and centers. A deeper analysis of the top-performing teams approaches reveals that style and photometric augmentations (contrast, blur, sharpness, etc.), known for their ability to induce significant intensity distribution variations, could be pivotal for enhancing model generalization in fetal MRI. This concept aligns with previous research into generalizable cardiac structure segmentation [38], [39]. Importantly, a potential trade-off between in-domain and OOD data generalization should be acknowledged [40]. For instance, the *NVAUTO* team, which scored first place in the in-domain data performance, did not use any specialized domain generalization techniques, yet fell to fourth place for OOD data. Notable in *NVAUTO*’s solution is that they used ensembling with 15 models. Conversely, *FIT_1*, initially third for in-domain data, rose to first place in the overall ranking, underscoring the indispensability of domain generalization in the development of robust image segmentation models. *FIT_1* also integrated ensembling in their solution, but with 5 models, plus an additional model that handled post-processing. It would be interesting to test if *FIT_1*’s would improve if they had also used a 15-model ensemble strategy, especially considering the time and energy it takes to train such a large number of models.

Interestingly, the performance metrics of the OOD images for some algorithms were not worse than the metrics for the in-domain images. Fig. 4 shows that the range of evaluation metrics for the in-domain results was much larger than the OOD results. This is primarily driven by the quality of the fetal brain reconstructions, as the average quality ratings of the OOD datasets (UCSF: 2.33; CHUV: 2.35) were higher than the average in-domain dataset quality ratings (Kispi: 2.18, Vienna: 1.95). Therefore, the quality of the fetal brain reconstructions played a large role in the success of the automatic segmentations.

### B. Overview of Top Three Teams

The top scoring teams all incorporated explicitly domain-robust solutions for the multi-site task. Teams *FIT_1* and *Bluebrune* both incorporated domain generalization strategies into their networks. Specifically, *FIT_1* used Painter by Numbers for style transfer training and *Bluebrune* used a domain adversarial approach in their training strategy. Transformer models did not seem to considerably help with domain generalization in our challenge data, as the highest-scoring team with a transformer model was *Blackbean*, who ranked 5^th^ and used a transformer model (ViT) as well as an nnU-Net. The results of the FeTA22 challenge indicate that existing model architectures and specialized data augmentation strategies can be used successfully to generalize segmentation networks. Short descriptions of the top three submissions can be found in the following sections, and the complete algorithm descriptions can be found in [16].

#### 1) *FIT_1*: Team *FIT_1*used a three-step process.

Firstly, they used data-augmentation-based domain generalization, followed by network ensembling, and an output-level denoising autoencoder that corrects implausible predicted segmentations [41]. *FIT_1* used nnU-Net as the network framework [36], and they trained five different models, each with their own data augmentation strategy. Model 1 used the default nnU-Net data augmentation steps. Model 2 added bias-field augmentation, an MR-specific data augmentation step. Model 3 adds style augmentation to the default nnU-Net and random bias field augmentations. Model 4 uses photometric augmentation, and model 5 uses MR-specific motion artifacts from moving subjects, simulated by TorchIO. These 5 networks were then ensembled based on average logit predictions. The final post-processing step is a rule-based post-processing with a denoising autoencoder (DAE), which takes the ensembled result as an input, and outputs a refined segmentation. The DAE model was trained using a self-generated dataset with noisy segmentations, and randomly dropping out features. The DAE was found to improve the segmentations when the input was poor, therefore the DAE step was only utilized with the large changes to the predictions occurred. In the training process, they generated three synthetic datasets for validation based on 24 cases, using default nnU-Net augmentation, random style augmentation [42], and bias-field augmentation [38].

#### 2) Bluebrune:

Team *Bluebrune* utilized a domain adversarial approach [43] to train this network, with nnU-Net as the framework. Their method involves two steps: a 3D nnU-Net segmentation network [36], followed by a domain discriminator network. The goal of the discriminator is to determine which site the input originates from (as there were two sites in the training data). They trained two different discriminator models, the first of which takes the outputted feature map before the soft-max layer from the 3D nnU-Net segmentation network as input in order to learn domain invariant features. The second discriminator takes the outputted feature map from the bottleneck layer of the Segmentation network as input in order to learn domain invariant features in the U-Net’s encoder. The discriminator networks output domain-class labels, and there is a gradient reversal layer inserted just before the discriminator networks, ensuring that the gradient passing to the segmentation networks is negative during backpropagation, ensuring adversarial network training. The two networks are trained separately. The discriminator networks are only used for training, and the two nnU-Net segmentation networks are used for inference and are combined with the softmax prediction average. They used standard nnU-Net preprocessing and data augmentation steps.

#### 3) FMRSK:

Team *FMRSK* took on a semi-supervised approach to train the networks. Firstly, they trained a standard 3D U-Net (using the MONAI framework [37]) to perform brain extraction. Next, they reviewed and rated the training data, scoring each case based on the quality of the labels. They then used the high-quality labels to train an initial Attention U-Net (again using the MONAI framework), and created labels with this network for the remaining cases. Manual corrections were made on these predicted labels. This iterative process was repeated three times. They utilized standard data augmentation steps, plus MR Spikes, bias fields, and random intensity shifts. They used an external dataset, using 19 developing Human Connectome Project (dHCP) neonates [44] who were scanned between 26.6–32.4 weeks, as well as a spina bifida atlas [45]. For training the final dataset, they used both the brain-extracted dataset and a version of the dataset with more of the surrounding structures, as well as flipping. They trained two attention U-Nets and averaged the predictions from the two models.

### C. Individual Labels

The top team (*FIT_1*) performed extraordinarily well across all labels, ranking first for four out of seven labels. The rankings of the other top teams were not as stable when looking at the individual fetal brain tissue labels, and no pattern could be found. As in the global rankings, the OOD volumes typically had better evaluation metrics for each individual label, apart from the ventricles and brainstem. It is uncertain why these two labels trended differently when compared to the remaining labels. The GM remains challenging to segment, which supports the importance of maintaining topology in automatic segmentations. The deep GM and brainstem were more challenging labels to segment, likely as the structures are not as clearly defined as there is not a strong demarcation and the difference in intensity from surrounding structures is reduced (unlike in structures such as the ventricles).

### D. Topological Analysis

In the analysis of topology as an evaluation metric, we demonstrated the importance of considering topology in the assessment and comparison of automatic segmentation methods. In the TIR algorithms ranking, the inclusion of a topology-based metric did not drastically change the final results, although minor updates are observed, and the across-tissue reliability of FMRSK in topological accuracy was rewarded.

## V. CONCLUSION

Our first multi-centric fetal brain segmentation challenge has demonstrated that overall, automated fetal brain segmentation has improved since the first FeTA Challenge [11]. However, there is still room for improvement in the segmentation of certain structures, especially in the cortical GM.

Despite the new challenges relating to generalizability, algorithms and training strategies have not changed drastically since FeTA 2021. All entries used deep learning and primarily 3D architecture. nnU-Net remained a popular and effective tool for medical image segmentation, and the most popular loss functions were the DSC loss and cross-entropy loss, or a combination of the two. Extensive data augmentation strategies are an integral aspect of training, and the addition of external datasets did not lead to better results. As with FeTA 2021, the top performing teams demonstrated a plateau in performance, likely due to the quality of both the SR algorithms and the quality of the manual segmentations.

Our challenge has demonstrated that the inclusion of just one additional institution (Vienna) into the training dataset, algorithms can improve their generalizability. Future research directions should focus on enhancing the generalizability of the methods, including the emerging low-field fetal MRI acquisitions [46], [47], or exploring federated learning approaches. In that context, conducting a more comprehensive evaluation of the impact of data augmentation and possible biases due to SR reconstruction methods would be very valuable. Furthermore, addressing challenges associated with inaccurate voxel-wise annotations and establishing standards of minimal image quality requirements [48], [49], [50] should be a priority. These endeavors would be crucially important to increase the clinical acceptance of automated fetal brain MRI segmentation.

## Figures and Tables

**TABLE I:** Training and Testing Dataset Properties from all Imaging Centers

	Testing Domain	Institution	Scanner	N	Super-Resolution Method	Resolution (mm^3^)	TR/TE	Gestational Age range (weeks)
Training	In-Domain	Kispi	GE Signa Discovery MR450/MR750 (1.5T/3T respectively)*	80	MIALSRTK (n=40) irtk-simple (n=40)	0.5×0.5× 0.5	TR: 2000–3500ms, TE: 120ms (minimum)	20.0–34.8
		Vienna	Philips Ingenia/Intera (1.5T); Philips Achieva (3T)*	40	NiftyMIC** (n=40)	1.0×1.0×1.0	TR: 6000–22000ms TE: 80–140ms	19.3–34.4
Testing	In-Domain	Kispi	GE Signa Discovery MR450/MR750 (1.5T/3T respectively)*	40	MIALSRTK (n=20) irtk-simple (n=20)	0.5×0.5×0.5	TR: 2000–3500ms, TE: 120ms (minimum)	21.3–34.6
		Vienna	Philips Ingenia/Intera (1.5T); Philips Achieva (3T)*	40	NiftyMIC** (n=40)	1.0×1.0×1.0	TR: 6000–22000ms TE: 80–140ms	18.1–35.0
	Out-of-Domain	CHUV	Siemens MAGNETOM Aera(1.5T)	40	MIALSRTK (n=40)	1.125×1.125×1.125	TR: 1200ms, TE: 90ms	21.0–35.0
		UCSF	GE Discovery MR750/MR750W (3T)	40	NiftyMIC** (n=40)	0..8×0.8×0.8	TR: 2000–3500 ms, TE: 100 ms (minimum)	20.0–35.1

* The training dataset contained data from both 1.5T and 3T scanners. However, which cases belonged to which scanner were not provided to the participants as it was part of the data anonymization process. Therefore, the breakdown of number of cases per scanner is not provided here.

** When the NiftyMIC algorithm was used, the image included the maternal tissue. The brain mask generated automatically by the algorithm was not used. Therefore, the NiftyMIC cases contained more maternal tissue than the fetal brains reconstructed with the MIALSRTK and irtk-simple algorithms.

**TABLE II:** FeTA 2022 Team Overview

Rank	Team Name	Architecture	Training Strategy	Loss Function	Post-processing	Augmentation	External Datasets

1	FeTA-ICL-TUM (FIT) – nnU-Net (FIT_1)	nnU-Net	Ensemble of 5 different models	CE and soft Dice	Ensemble + rule-based denoising autoencoder post-processing	(i): Default nnU-Net augmentation; (ii): (i) + random bias field; (iii): (i) + style augmentation, random bias field; (iv): (i) + photometric augmentation; (v): (i) + motion artifact	‘Painter By Numbers (PBN) for style transfer training in (iii)
2	BlueBrune*	nnU-Net	Data Split: 80/20; (i) tissue segmentation network; (ii) domain adversarial approach	CE and dice	ensemble (2 models)	default nnU-Net augmentation	No
2	FMRSK*	(i) 3D U-Net (ii) Attention U-Net (MONAI)	(i) brain extraction; (ii) tissue segmentation	Dice and CE (MONAI)	Average prediction of 2 models	motion artifact, MR spike, Bias field, affine transform, noise, blurring, gamma, random intensity shift	19 dHCP neonates, Spina bifida atlas
4	NVAUTO	SegResNet	5-folds CV	Dice Focal from MONAI	ensemble on average prediction (15 models)	normalize to zero mean and unit standard deviation	No
5	Blackbean	(i) nnU-Net, (ii) ViT-Adaptor	Data Split: 100/0; (i) two models, (ii)Test-time augmentation	Dice and BCE	Ensemble on test-time augmented (softmax mean)	default nnU-Net augmentation	No
6	symsense	nnU-Net	5-folds CV	modified Generalized Dice and CE	None	Default nnU-Net augmentation, brightness transform, contrast transform, zoom, warping, GIN-IPA	40 early neonatal dHCP
7	FeTA-ICL-TUM (FIT) – SWINUNETR (FIT_2)	(i) Synthstrip, (ii) Swin UNETR (MONAI)	(i) skull stripping; (ii) tissue segmentation	weighted CE and soft Dice	Resampling to original image	flipping, rotation, affine + elastic transformation, noise, blur, gamma, ghosting, spike, motion, bias, blur, anisotropy	neonate subjects of the dHCP
8	DBC Pasteur	U-Net	Ensemble of 3 2D networks (ax/sag/cor) Data split 80/20%	CE	Ensemble (3 models) majority vote	Noise, blur, 2D rotations + translations + flip, zoom	Atlas Gholipour + Atlas Scrag
9	Dolphins	(i) 3DResUNet (coarse), (ii) nnU-Net (fine)	5-folds CV, keep the one with best validation scores	BCE+Dice	None	Flipping, random gamma	No
10	fudan_zmic	BayeSeg (based on nnU-Net)	5-folds CV	CE, Dice, and weighted variational	Ensemble (5 folds cv)	cardiac cutmix augmentation to enrich the background, and nnU-Net augmentations	ACDC dataset for cutmix
11	hilab	(i) nnU-Net, (ii) residual 3D U-Net	(i) coarse multi-class model, (ii) seven fine single class models	CE and Dice loss	Ensemble of stage (ii).	rotation and scaling, Gaussian noise and blur, brightness and contrast adjustment, simulation of low resolution, gamma augmentation, mirroring	No
11	Neurophet	3D U-Net	3 models trained; probability-based sampling method to focus network	Dice, CE (custom weight)	All 3 models used, non-zero voxels measured, volumes compared	spatial (horizontal flip, rotation, affine transform) and intensity (gaussian blur)	No
13	Sano	Swin UNETR	5-folds CV	CE and Dice	Ensemble learning (5 models)	cropping, random zoom, random rotation, random gaussian noise, random adjust contrast, random flip on each axis	No
14	Uniandes	ROG (from MSD)	2-folds CV	Dice and CE	closure and opening of grays in segmentation map with structuring element	Spatial Transform (random rotation and scaling), Mirror Transform and gamma correction.	No
15	xinlab-scut-iai-ahu	(i) DynUNet, (ii) Swin Transformer, (iii) SwinUNETR	5-folds CV; (i) brain extraction, (ii) Domain generalization stage, (iii) tissue segmentation	(i) Dice, (ii) Contrastive, CE, L2 (iii) Dice	argmax operated results of sliding window patches with 0.5 overlap	orientation change, spacing, cropping, flipping, rotation	No
16	deepsynth	U-Net, SynthSeg	Training on synthetic, fine tuning on real T2w (dHCP, FeTA)	Mean dice score	None	Synthetic MRI from dHCP label-maps with SynthSeg	80 youngest dHCP subjects
17	ajoshiusc	R50-ViT: combo of ResNet-50 and ViT, transUNet	Data Split: 75/5 subjects.	CE, robust CE based on beta divergence	None	None	No

BCE: Binary Cross-Entropy, CE: Cross-Entropy, CV: cross-validation, dHCP: developing Human Connectome Project, MSD: Medical Segmentation Decathlon

**TABLE III:** Final Rankings and Results of the FeTA 2022 Challenge. In Addition, the Separate Rankings for the In-Domain Datasets (Kispi, Vienna) and the Out-of-Domain Datasets (CHUV, UCSF) are Shown

Global ranking	Team Name	Global Average DSC	Global Average HD95	Global Average VS	In-Domain Ranking	Out-of-Domain Ranking
1	FIT_1	0.816 ± 0.11	2.347 ± 2.51	0.910 ± 0.12	3	2
2*	Bluebrune	0.812 ± 0.11	2.377 ± 2.55	0.908 ± 0.11	6	3
2*	FMRSK	0.808 ± 0.11	2.395 ± 2.94	0.920 ± 0.10	9*	1
4	NVAUTO	0.810 ±0.13	2.608 ± 3.30	0.915 ± 0.12	1	4*
5	Blackbean	0.812 ± 0.12	2.506 ± 3.66	0.909 ± 0.11	7	4*
6	Symsense	0.813 ± 0.12	2.660 ± 6.81	0.907 ± 0.12	4	4*
7	FIT_2	0.798 ± 0.11	3.421 ± 5.51	0.913 ± 0.10	2	7
8	Institute Pasteur (DBC)	0.789 ± 0.13	2.387 ± 1.97	0.901 ± 0.12	8	8
9	Dolphins	0.806 ± 0.12	4.521 ± 11.87	0.905 ± 0.12	9*	9
10	Fudan_zmic	0.788 ± 0.13	4.720 ± 7.58	0.903 ± 0.12	11	10
11	Hilab	0.774 ± 0.13	13.008 ± 14.84	0.887 ± 012	12*	12
12	Neurophet	0.739 ± 0.22	10.288 ± 35.54	0.844 ± 0.25	5	14
13	Sano	0.709 ± 0.22	7.171 ± 12.76	0.817 ± 0.23	14	11
14	Uniandes	0.652 ± 0.22	11.366 ± 27.61	0.814 ± 0.23	12*	13
15	Xinlab-scut	0.494 ± 0.29	23.150 ± 20.66	0.731 ± 0.22	15	15
16	Deepsynth	0.433 ± 0.34	36.653 ± 62.13	0.604 ± 0.38	16	16
17	Ajoshiusc	0.319 ± 0.33	56.598 ± 75.01	0.480 ± 0.38	17	17

* Tied

**TABLE IV:** Topology (a) and Global (b) Rankings of the Submissions. (a) Betti Number Errors (BNE) Per Dimension and Overall. (b) Comparison of the FeTA Challenge 2022 Ranking and the Topology-Integrative Ranking (tir). Top 3 Submissions are Shown in Bold

Team Name	(A) Topology			(B) Global		

	k-dim BNE			BNE	TIR	FeTA

	BNE0	BNE1	BNE2			

ajoshiusc	16	15	16	16	17	17
Blackbean	3	3	4	3	3	5
BlueBrune	2	2	3	2	2	2
Deepsynth	17	16	17	17	16	16
Dolphins	5	7	7	5	8	9
FIT_1	1	1	2	1	1	1
FIT_2	7	8	5	6	7	7
FMRSK	9	4	13	9	4	3
Fudan_zmic	8	10	9	10	10	10
Hilab	10	11	1	8	11	11
Institut_Pasteur_DBC	11	12	11	12	9	8
Neurophet	14	14	15	14	12	12
NVAUTO	6	6	8	7	5	4
Sano	13	13	12	13	13	13
symsense	4	5	6	4	6	6
Uniandes	12	9	10	11	14	14
Xinlab-scut-iai-ahu	15	17	14	15	15	15

**TABLE V:** Topology (BNE) Ranking of the Submissions Per Tissue Class and on Average

Team Name	eCSF	GM	WM	Ventricles	Cerebellum	deep GM	Brainstem	Average

Ajoshiusc	10	15	5	17	16	17	14	13.4
Blackbean	4	4	1	2	4	6	2	3.3
BlueBrune	5	7	2	3	1	1	3	3.1
Deepsynth	14	16	7	15	17	16	17	14.6
Dolphins	7	8	3	8	2	5	6	5.6
FIT_1 (nnU-Net)	2	5	4	1	3	2	1	2.6
FIT_2 (SWINUNETR)	3	2	6	10	8	7	5	5.8
FMRSK	12	10	9	9	6	8	10	9.1
Fudan_zmic	9	11	8	11	9	9	8	9.3
Hilab	1	6	13	5	11	12	12	8.6
Institut_Pasteur_DBC	15	13	11	12	7	10	11	11.3
Neurophet	16	12	14	14	15	14	16	14.4
NVAUTO	11	1	12	6	5	3	7	6.4
Sano	13	14	15	13	12	13	9	12.7
symsense	8	9	10	4	10	4	4	7.0
Uniandes	6	3	16	7	14	11	15	10.3
Xinlab-scut-iai-ahu	17	17	17	16	13	15	13	15.4
